# The Doctors’ Weekly

**Published:** 1892-02

**Authors:** 


THE DOCTORS' WEEKLY, a bright, spicy and newsy weekly journal, published at New York, by Dr. Ferdinand King, has been received, and is a new departure as to form, being in fact, an eight-page newspaper, devoted to the interests of the profession of New York City and vicinity.

---

WE ARE in receipt of a case of Dr. G. W. Powell's Rectal Instruments, and can say that they are a most valuable acquisition to any doctor's instrument case, being neat, well made, and admirably adapted to work of that particular kind.

---

### LUMBAGO.

Dr. Lyman Watkins says (*Med. Gleanor*) that ten drops of the tincture of gelsemium every four hours will almost invariably relieve that painful condition, or backache, commonly called lumbago.—*Med. and Surg. Reporter.*

